# Normal values for wall thickening by magnetic resonance imaging

**DOI:** 10.1186/1532-429X-11-S1-P61

**Published:** 2009-01-28

**Authors:** Joey Ubachs, Einar Heiberg, Katarina Steding, Håkan Arheden

**Affiliations:** Cardiac MR Group, Dept. Clinical Physiology, Lund University Hospital, Lund, Sweden

**Keywords:** Wall Motion, Left Ventricular Mass, Wall Motion Abnormality, Fractional Wall, Area Assessment

## Purpose

The aim of this study was therefore to describe normal values for myocardial wall motion using steady state free precession MRI.

## Introduction

Regional left ventricular dysfunction is a major consequence of myocardial ischemia. Accurate quantitative analysis of wall motion is therefore essential for risk area assessment. In order to assess wall motion abnormalities, normal values need to be established. Although previous studies exist, to our knowledge however, no absolute regional values for wall thickening have been published using MRI steady state free precession pulse sequence (SSFP). It has been shown that there is a statistical difference in left ventricular mass between SSFP and the older gradient echo pulse sequences.

## Methods

Fifty healthy, non-athletic subjects (age: 35 ± 11, M/F: 30/20) were included in this study. Short-axis SSFP cine images were taken and absolute wall thickening and fractional wall thickening were assessed after manual tracing of the endocardial and epicardial borders. As an internal quality check, tracings were considered correct when difference in left ventricular mass between end-diastolic and end-systolic tracings was less than 2%. Normal values for wall thickening were calculated and converted into the American Heart Association 17-segment model. The most basal slice included in the analysis, was the first slice below the membranous septum in end-systole. The most apical slice was excluded.

## Results

**Figure 1:**
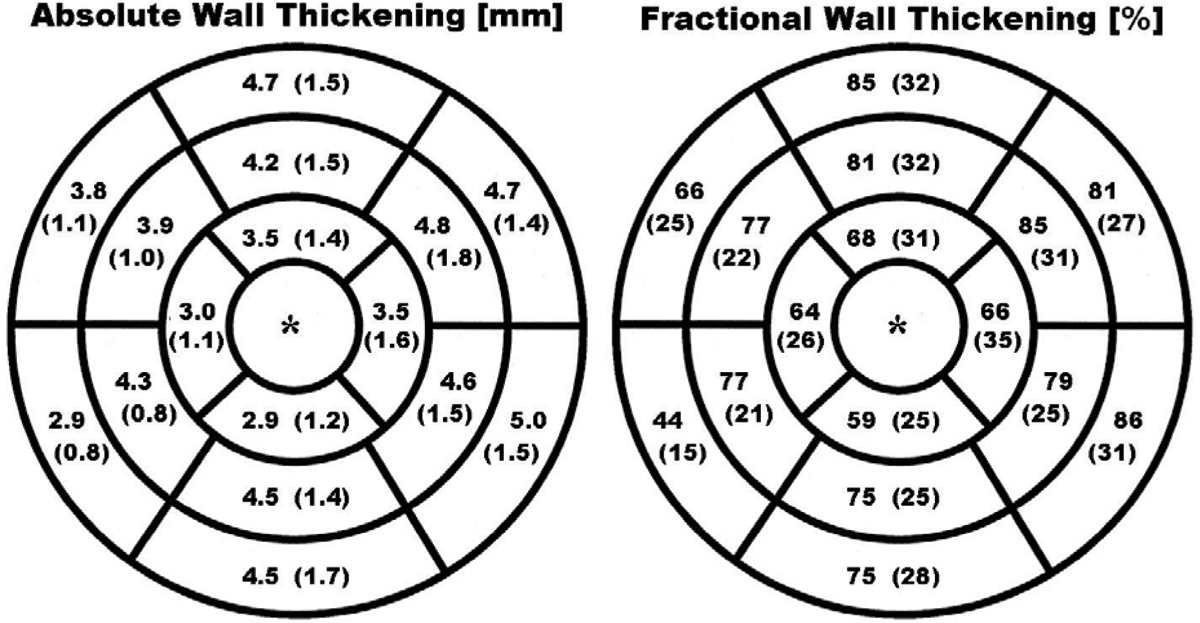
**Measurements of absolute wall thickening (left panel) and fractional wall thickening (right panel) are displayed in a 17-segment model**. Wall thickening measurements in the most apical slice is not meaningful in short-axis images and were therefore left out*. Absolute wall thickening is expressed in millimetre (standard deviation), fractional wall thickening in percentage (standard deviation).

Mean normal values for absolute wall thickening were 4.3 ± 1.4 mm (basal slices), 4.4 ± 1.4 mm (mid-ventricular slices), and 3.2 ± 1.4 mm (apical slices). Mean normal values for fractional wall thickening were 73 ± 31% (basal slices), 79 ± 26% (mid-ventricular slices), and 64 ± 30% (apical slices). Between men and women, there was no significant difference in absolute wall thickening (p = 0.06), but a significant difference in fractional wall thickening (p = 0.003). Figure 1.

## Conclusion

The present study presents normal values for wall thickening derived from short-axis MRI. These normal values can be used as reference for quantitative assessment of wall motion abnormalities in various cardiac disease states.

